# Precision ventral extraction of implanted mouse brains: A protocol

**DOI:** 10.1016/j.xpro.2026.104677

**Published:** 2026-07-08

**Authors:** Lyla El-Fayomi, Michael Bergamini, Derek van der Kooy

**Affiliations:** 1 Krembil Research Institute, Toronto Western Hospital, Toronto, ON M5T 0S8, Canada; 2 Denver Health Medical Center, Denver, CO 80204, USA; 3 Department of Molecular Genetics, University of Toronto, Toronto, ON M5S 3E1, Canada

**Keywords:** Health Sciences, Model Organisms, Neuroscience, Biotechnology and bioengineering

## Abstract

Neural implants are critical for modern mouse studies; however, permanent fixation of fibre optics or electrodes can render brain removal for histology particularly challenging. Frequent complaints include implant damage and tissue distortion. Here, we outline steps for transcardial perfusion and cranial isolation, then describe a precise dissection that facilitates brain extraction from the skull’s ventral aspect. We further detail post-fixation and cryoprotection steps. Our approach is anchored in clear anatomical landmarks to ensure minimal tissue trauma, clean separation, and reproducibility.

For complete details on the use and execution of this protocol, please refer to El-Fayomi et al*.*^1^

## Highlights

• Procedures for mouse transcardial perfusion
• Instructions for implanted brain extraction via ventral dissection
• Optional post-fixation and cryopreservation steps

## Abstract (editor highlights)

Publisher’s note: Undertaking any experimental protocol requires adherence to local institutional guidelines for laboratory safety and ethics.

## Graphical abstract

**Figure:**
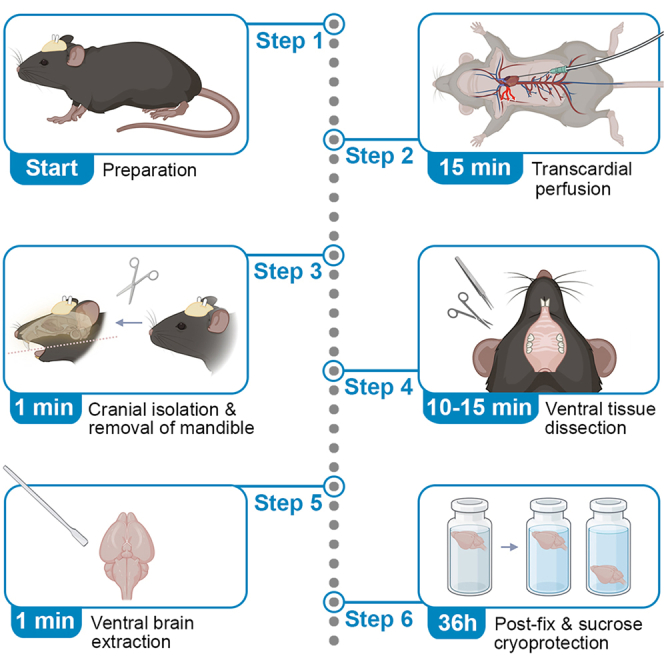

## Teaser

Neural implants are critical for modern mouse studies; however, permanent fixation of fiber optics or electrodes can render brain removal for histology particularly challenging. Frequent complaints include implant damage and tissue distortion. Here, we outline steps for transcardial perfusion and cranial isolation, then describe a precise dissection that facilitates brain extraction from the skull’s ventral aspect. We further detail post-fixation and cryoprotection steps. Our approach is anchored in clear anatomical landmarks to ensure minimal tissue trauma, clean separation, and reproducibility.

## Before you begin

Chronic neural implants, including fibre optic cables and electrophysiological probes, are commonplace in contemporary mouse studies. However, permanent fixation^2^ can render brain removal for histology particularly challenging, especially if implants enter at an angle. Dorsal dissection approaches often lead to implant breakage within the brain, in addition to tissue distortion that may complicate placement confirmation. Such issues also affect image quality for publication purposes.

Here, we present a concise protocol for brain fixation, dissection and extraction from the ventral aspect of the skull, anchored in clear anatomical landmarks throughout the oral cavity^3^ and bone.^4^ We designed this technique to ensure reproducibility within and between research groups, minimize tissue trauma, and allow for clean separation.

We use this protocol along with a peristaltic pump system to circulate the saline and fixative (4% formaldehyde/10% formalin solution), but our protocol is also compatible with manual perfusions using syringes. You may opt for other fixatives with similar properties and safety indications.

Importantly, this protocol has not been attempted on fresh, unfixed mouse brains. Therefore, please be advised that, to our knowledge, fixation is critical for the results we produce here. However, following brain extraction from the skull, researchers may need to amend the final steps (post-fixation, sucrose cryoprotection, etc.) accordingly, depending on their experimental requirements.

Last, we have successfully applied this protocol to neural implants including multielectrode and optetrode drives, plus bilateral Ø200 μm fibre optic implants lowered atop regions at 10^o^–20^o^ angles.^1^ We have not specifically validated this protocol for other types of implants, such as larger lenses for live imaging.

### Innovation

To our knowledge, an anatomically precise protocol for ventral brain extraction has not been established until now. This is an unmet need within our field, given the limitations of dorsal brain dissections outlined above. Presenting researchers with an option for extraction built on a foundation of textbook cranial anatomy and well-defined, identifiable structures will inspire greater confidence in adoption of the approach, stronger reproducibility, and fewer errors.

### Institutional permissions

All animal use procedures were approved by the University of Toronto Animal Care Committee, in accordance with the guidelines of the Canadian Council on Animal Care. Approval from your institutional committee will be required to perform this protocol as well.

### Workstation preparation

**Timing: Variable**

This protocol should be carried out in a fume hood. Users must don proper PPE corresponding to the chemical and biological hazards presented, and must follow the relevant safety guidelines in place at their institutions.

1. Fill the appropriate number of scintillation vials (or other suitable specimen jars) with 20 mL of fixative solution.
  a. Label them in advance with corresponding animal ID codes and keep them on ice.
  b. If the brain contains light-sensitive molecules (e.g., fluorescent markers), wrap the vials in foil and secure with tape.
2. Aliquot the correct volume of saline and fixative you will require for the perfusion session in secure containers (e.g., autoclaved glass bottles) and keep them on ice.

***Note:*** Always prepare more than you need; for instance, 30% of your calculated total serves as an adequate buffer for leaks, equipment malfunctions, or other unexpected events.

**Figure 1:**
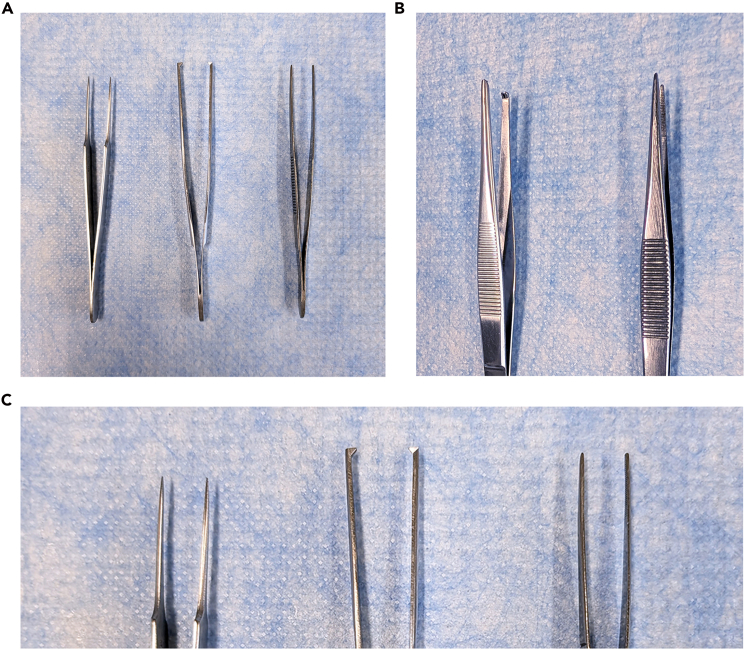
Forceps required for dissection (A) A side view of the three different forceps to be used throughout the ventral dissection protocol. Left, fine forceps (e.g., Dumont forceps). Middle, tissue forceps with teeth. Right, straight serrated forceps. (B) Magnified, angled view of forceps’ defining features. Left, toothed tissue forceps. Right, straight serrated forceps. (C) Magnified side view of forceps.

3. Sterilize all dissection tools (see Figures 1 and 2) and place them on a clean surface in an easily accessible area.

**Figure 2:**
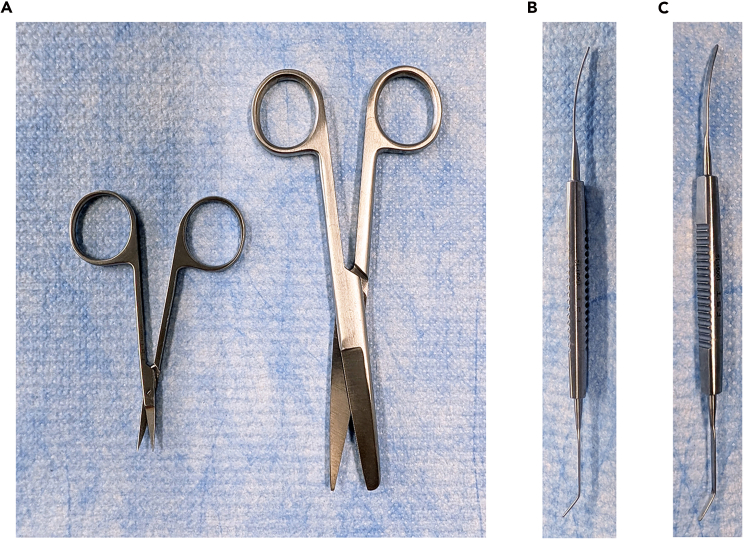
Scissors and micro spatula required for dissection (A) Left, iris scissors. Right, sharp/blunt operating scissors. (B) Side view of double-sided micro spatula. (C) Angled view of double-sided micro spatula.
4. Clean the outside of the peristaltic pump tubing.

**CRITICAL:** Ensure tubing for saline and tubing for fixative solution are clearly labelled and kept separate.

5. Curl the tubes into their respective bottles of solution and pull Parafilm over the top of each one.
  ***Note:*** This helps avoid aerosols, protect the contents, and hold the tubing in place.
  a. Keep these bottles in an ice box as you work with them.
  b. Ensure that the tips of the tubing are fully submerged in liquid and are as close to the bottom as possible.
6. Attach needles to the opposite ends of the tubes and poke the needle tips through the Parafilm on their respective bottles to keep the sharps at a safe distance away from you when not in use.
  ***Note:*** This is especially important when using a peristaltic pump, as on certain models, both solutions will run at once. Pointing the needle that is not in use into the bottle through the Parafilm will prevent spills and waste.
  a. Test the pump to ensure it is moving fluids as expected prior to beginning.
    i. At minimum, you must clear all air from the tubing prior to use.
7. Pre-load syringes with sodium pentobarbital (or other appropriate, institutionally-approved agent) for euthanasia.
8. Have your first scintillation vial out in front of you, which you will be using to collect the brain following extraction.

***Note:*** Keep it open but at a distance, such that it will not accidentally be knocked over.

9. Create a perimeter around your immediate perfusion area (where the mouse will be) by rolling up a few pieces of paper towel and taping them down to your perfusion surface (styrofoam, metal, etc.).

***Note:*** This contains the lateral spread of blood and chemicals to a focused area.

**CRITICAL:** Ensure you have determined how best to collect the chemical and blood run-off from the procedure in such a way that is compliant with your facility’s hazardous materials policies.

10. Have four pieces of tape ready to fix the animal in the supine position.
11. Prepare folded, disposable absorbent materials soaked in a compatible disinfectant cleaner to wipe blood and tissue off your tools in between steps, as needed.
12. Locate the correct (chemical contamination) animal carcass disposal bag and keep one open adjacent to your perfusion surface (also within the fume hood) for biological dissection waste generated as you work.

***Note:*** Always follow policies for safe containment and disposal of the carcass at your facility.

13. Keep live mice at a significant distance from your station to minimize distress.

## Key resources table

**Table:** 

REAGENT or RESOURCE	SOURCE	IDENTIFIER
**Chemicals, peptides, and recombinant proteins**		

10% Formalin/4% Formaldehyde Solution	Millipore Sigma	#1004965000
DPBS 1X	ThermoFisher	#14190144
Euthanyl (Sodium Pentobarbital) - Discontinued	McGill University Comparative Medicine Animal Resource Centre	#381

**Experimental models: Organisms/strains**		

*Gad2*^*tm2(cre)Zjh*^/J (GAD2-IRES-Cre) ➢Adults (>12 weeks old), both males and females	Jackson Laboratory	Stock no.: #010802, RRID: IMSR_JAX:010802

**Other**		

Cole-Parmer Ismatec® Peristaltic Pump	Cole-Parmer Instrument Company	78017-00

## Step-by-step method details

### Transcardial perfusion of experimental mouse

**Timing: Approx. 15 min**

***Note:*** This section of the protocol will clear blood from the brain tissue and circulate a fixative chemical for preservation.

1. Take your first mouse out of the cage.
  a. Scruff it tightly and carefully inject the proper dosage of sodium pentobarbital I.P.

***Note:*** You must comply with your facility’s dosage guidelines for sodium pentobarbital.

**CRITICAL:** Do not re-shield the needle.

2. Release the animal back into its cage so that it is comfortable.
3. When the animal is unconscious, and the toe pinch reflex is *completely* absent, tape the mouse down in supine position.
4. Palpate the abdomen and search for the xiphoid process with your finger.
  a. Once you feel it, use tissue forceps to pinch it and pull upwards.
  b. With your other hand, use operating scissors (sharp-blunt configuration) to cut through the skin and the abdominal wall just below the rib cage.

***Note:*** The blunt side should be oriented toward the organs during this step.

5. Identify the diaphragm.
6. With the blunt end of the scissors always oriented toward the heart’s location, cut through the diaphragm.

**CRITICAL:** Avoid all other organs – damaging them might compromise the circulatory system and thus reduce the efficacy of saline and fixative runs.

7. Once the diaphragm is clear, make upward lateral cuts along the rib cage, creating more room to manoeuvre.

***Note:*** The heart should be visible at this point, and it should still be beating (although again, the animal should be entirely unconscious).

8. Use iris scissors to make a small incision in the right atrium.

***Note:*** Blood will flow outward and pool if this is done correctly.

9. Using tissue forceps, gently orient the heart such that the left ventricle is accessible.
10. Using the needle attached to your saline tube, pierce the left ventricle.

**CRITICAL I:** Confirm that the correct tube is in use (saline, not fixative).

**CRITICAL II:** Try not to poke through the septum by ensuring your needle is pointing toward the mouse's head in a horizontal plane – parallel to the septum – and by controlling your depth appropriately. Gently use your tissue forceps as leverage.

11. Once the needle is in the left ventricle of the heart, run the pump until 20–40 mL of saline have been circulated.

***Note I:*** If your needle has pierced the ventricle, it will swell with saline once the pump begins.

***Note II:*** The liver and skin will become dramatically more pale, given that the blood has been flushed from the system; this is an indicator of success.

12. Stop the pump before removing the needle from the heart, but make a note of the entry/exit point in the tissue to minimize the number of perforations created (which are technically leakage points, if not plugged).

***Note:*** Remember to insert the saline needle back into the Parafilm.

13. Insert the fixative needle into the left ventricle at the entrance site created previously.
14. Run the pump until 30–40 mL of fixative have been circulated.

***Note:*** It is normal for the tail and limbs to twitch during this process.

15. Stop the pump before removing the needle.

***Note:*** You should feel a change in the rigidity of the heart tissue as the needle is removed; it will be significantly firmer.

16. Test the animal’s tail and limbs for rigidity to ensure proper fixation has been achieved.

***Note:*** Your transcardial perfusion is now complete. Next, you will remove the brain for histology.

### Ventral brain dissection and extraction

**Timing: Approx. 10–15 min**

17. Use operating scissors to make a cut above the shoulders and behind the ears to sever the head.
18. Insert the sharp blade of your operating scissors into the oral cavity and cut through soft tissue and bone on both sides *(∼2 cuts per side).*
  a. You should now be able to peel away the mandible and attached tissues, including the tongue (which will be entirely visible).
19. Cut away the ears and extra skin *(∼2***–***3 cuts).*
20. Make two large cuts on either side of the head, removing as much soft tissue as possible *(∼1 cut per side)*.
21. Cut away the molars on each side, as these are sharp and can hurt you *(∼1 cut per side)*.
  a. You may opt to cut away the upper incisors as well.
22. Insert iris scissors into the foramen magnum and make lateral cuts in the skull, framing the tissue toward the soft palate *(∼2***–***3 cuts per side)*.
  **CRITICAL:** Ensure iris scissors are flush to the bone, opposite to the direction of the brain; scissors should snap outwards with each cut as a result of pressure applied away from the midline.
  a. Using serrated forceps, you should now be able to peel off the posterior region of the skull (from the occipital bone toward the basisphenoid bone).
23. Continue with lateral cuts, framing what remains of the soft palate, noting a change in the slope of the skull *(∼2***–***3 cuts per side).*
  a. You may now peel away what is left of the basisphenoid bone toward the palatine bone.

***Note:*** You should see the optic nerves and have access to the anterior part of the brain; the only hidden structures at this point should be the olfactory bulbs. You may continue making framing cuts farther anteriorly along the hard palate if you wish to collect the olfactory bulbs as well.

***Note:*** The skull will be curved around the brain, cupping it, if your cuts are at the correct position.

24. Peel all edges of the skull away with fine forceps so that the brain can come out freely.

***Note:*** This is especially important around the cerebellum; there are two lateral bony structures at the junction between the cerebellum and the rest of the brain, so make sure to snap these away with fine forceps without damaging the tissue.

25. Using a micro spatula, loosen/separate the brain from the skull around the perimeter of the structure, taking great care not to damage the tissue and maintain contact between metal and bone where necessary.

***Note:*** It is very easy to slice through the brain with this tool if moving too quickly.

26. Turn the skull upside down over your labelled scintillation vial.
27. Gently insert the spatula deeper and deeper against the skull, symmetrically encouraging the brain away from the implants.

***Note:*** Gravity will do half of the work here. The brain will eventually loosen from the implants and fall into the vial.

**CRITICAL:** Tugging too hard will stretch or tear the brain, so this is a very delicate process. Also, ensure your motions are symmetrical so as not to apply disproportionate force to one side, creating shear stress on the implant tracts.

28. Once the brain is removed, ensure implants are intact by looking at the inside of the skull and feeling for them with your scooping tool.
29. Change into clean gloves and close the vial before returning it to the ice bucket.
30. Document relevant information pertaining to procedure (fixation quality, volume of anaesthetic used, start and end time, any issues you may have encountered, etc.).
31. Repeat protocol as needed for each mouse.

### Optional follow-up steps from our histology workflow, subject to user customization

32. Once finished, store brains at 4 degrees Celsius for ∼12**–**16 h (post-fixation period).
33. Following post-fixation, transfer the brains into new scintillation vials containing a 30% sucrose solution for cryoprotection and note the time of transfer.
  a. Brains will initially float in this solution; when they have sunk, they are ready for cryosectioning.

***Note:*** We usually allow approximately 24h for this.

## Expected outcomes

**Figure 3:**
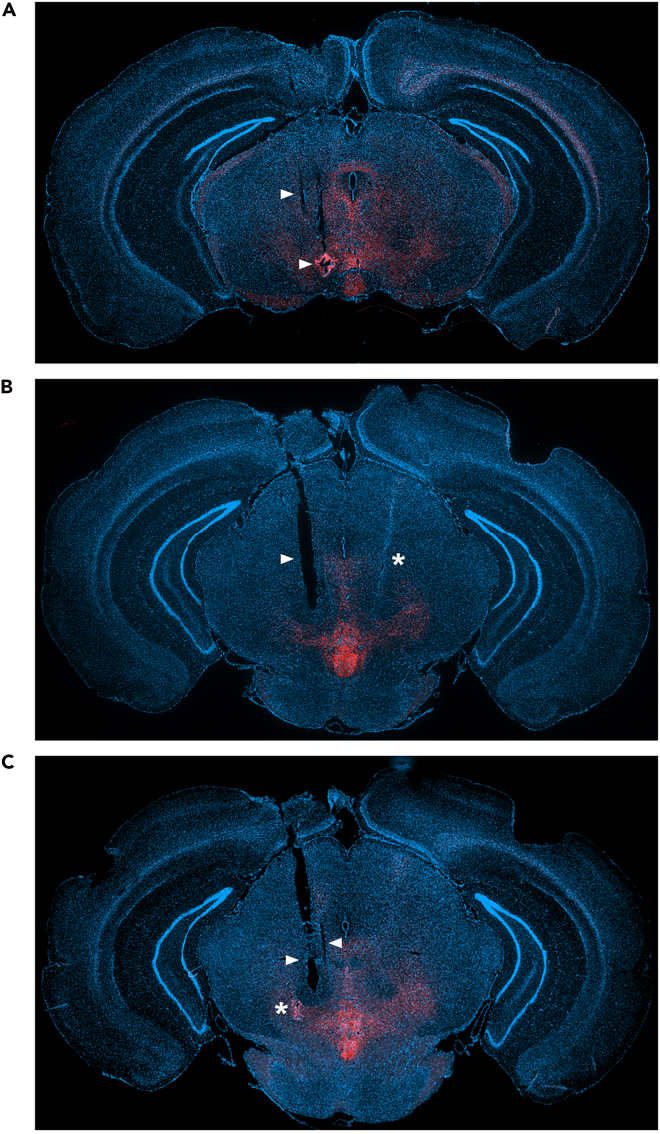
Ventrally dissected, optetrode-implanted mouse brain sections Coronal sections displaying implant tracts created by an optetrode brain implant (Neurotek-IT Inc., custom build). Blue fluorescence represents Hoechst (nuclei) and red fluorescence represents ChR2(E123A) opsin expression in GAD65+ (GAD2+) neurons. (A) Left marker, a portion of tetrode tract. Right marker, electrolytic lesion at the terminus of a tetrode that yielded usable cell recordings. (B) Left marker, fibre optic cable tract. ∗, injection tract gliosis. (C) Closely positioned tracts are still intact and identifiable. Left marker, fibre optic cable tract. Right marker, tetrode tract. ∗, the edge of a lesion from a tetrode not captured in this slice.

This protocol prepares the mouse brain for subsequent validation of implant placements and fluorescent marker detection via microscopy. After the above steps, we cryosection our extracted mouse brains, stain them with Hoechst (a nuclear dye), and coverslip them. Representative images are published in El-Fayomi *et al.*^1^ We include an additional, previously unpublished example here (Figure 3): Our protocol was successfully applied to the histological analysis of a mouse with an optetrode brain implant (Neurotek-IT Inc., custom build) targeted to the ventral tegmental area. The adult animal (GAD2-IRES-Cre) had received bilateral injections of a floxed opsin gene, AAV-EF1a-DIO-ChR2(E123A)-mCherry. The implant included a Ø200 μm fibre optic cable with tetrode bundles positioned around it. Electrolytic lesions were created at the tips of tetrodes that captured testable cell recordings. By removing the fixed brain from the ventral aspect of the skull, we were able to preserve the tetrode tracts (Figure 3A) and the fibre optic cable tract (Figure 3B) with little to no distortion. In fact, despite their close proximity, tetrode tracts did not merge with the fibre optic tract (Figure 3C); they are clearly separable.

It is important to note that when proper training is prioritized, a very high success rate is entirely realistic. Our group has never lost a brain due to the failure of this protocol, and none of our users have broken implants within the tissue. However, these statistics correspond to the following structures historically targeted by us^1^: The ventral tegmental area, the tegmental pedunculopontine nucleus, the dorsal raphe nucleus, and the nucleus accumbens. As previously mentioned, our implants were also angled at a maximum of 20^o^. There is a chance that the targeting of other structures might prove more challenging, depending on location and implant angling.

## Limitations

This protocol requires a high degree of animal handling and dissection experience; we recommend practice dissections on non-implanted mice, meaning brains that do not need to be collected for experiments. This will help researchers familiarize themselves with the external anatomical landmarks in the oral cavity (for example, hard palate versus soft palate), in addition to those of the skull, which may be less visible during initial dissection. Practice dissections will further allow researchers to learn the internal curvature of the skull and avoid slicing through the brain throughout the final extraction steps.

Great care should also be taken to avoid bringing contaminants (blood, extraneous tissue, hair) from the animal into contact with the specimen, further emphasizing the necessity for practice.

As previously mentioned, perfusion, post-fixation and cryoprotection steps may need to be amended, depending on the experiment’s unique histological requirements; however, the dissection segment (which is the core purpose of this protocol) would likely remain constant.

## Troubleshooting

### Problem 1

Fluid is coming out of the nose during saline or fixative perfusion (related to steps 10 and 13).

### Potential solution

The needle has infiltrated the wrong sector of the heart, and pulmonary backflow is occurring. Re-position the needle to ensure it is not too deep and is not angled – it should point straight up into the ventricle.

The flow rate on the pump may also be too high; consider reducing it.

### Problem 2

The animal’s toe pinch reflex will not disappear (related to step 3).

### Potential solution

The I.P. injection may not have been completed correctly, the euthanasia drug may be expired, or the dosage may be incorrect. Always ensure protocol users have received adequate training on injections, are checking expiry dates (plus setting corresponding reminders), and are calculating the correct volume of the drug to be administered based on the weight of the animal.

### Problem 3

The animal’s heart is no longer beating upon initial visualization (related to step 7).

### Potential solution

It is possible that the dose of sodium pentobarbital was too high, or the user did not proceed quickly enough. Dosing and speed should be priority adjustments on the next animal.

### Problem 4

The peristaltic pump is running, but no fluid is being expelled from the tube (related to preparation step 6a).

### Potential solution

Old tubing may sometimes become flattened over time, preventing liquid from passing through it; remove the tubes to check them, and replace if necessary.

## Resource availability

### Lead contact

Further information and requests for resources and reagents should be directed to and will be fulfilled by the lead contact, Dr. Lyla El-Fayomi (lyla.el.fayomi@mail.utoronto.ca).

### Technical contact

Technical questions on executing this protocol should be directed to and will be answered by the technical contact, Dr. Lyla El-Fayomi (lyla.el.fayomi@mail.utoronto.ca).

### Materials availability

This study did not generate new unique reagents.

### Data and code availability

This study did not generate/analyze datasets or code.

## Acknowledgments

This work was supported by a Canadian Institutes of Health Research Foundation Grant to D.v.d.K. (FDN-148407). We also thank the Division of Comparative Medicine at the University of Toronto. The graphical abstract was created in BioRender. El-Fayomi, L. (2026) https://BioRender.com/9jjfrpd.^5^

## Author contributions

L.E. and M.B. conceived the protocol. L.E. designed the protocol and wrote the original draft. L.E., M.B., and D.v.d.K. reviewed and edited the protocol.

## Declaration of interests

The authors declare no competing interests.
